# Monitoring underwater volcano degassing using fiber-optic sensing

**DOI:** 10.1038/s41598-024-53444-y

**Published:** 2024-02-07

**Authors:** Corentin Caudron, Yaolin Miao, Zack J. Spica, Christopher Wollin, Christian Haberland, Philippe Jousset, Alexander Yates, Jean Vandemeulebrouck, Bernd Schmidt, Charlotte Krawczyk, Torsten Dahm

**Affiliations:** 1 Laboratoire G-Time, Department of Geosciences, Environment, and Society, Université Libre de Bruxelles, Brussels, Belgium; 2 Department of Earth and Environmental Sciences, University of Michigan, Ann Arbor, MI USA; 3 GFZ German Research Centre for Geosciences, Potsdam, Deutschland Germany; 4 ISTerre, Université Grenoble Alpes, Université Savoie Mont Blanc, CNRS, IRD, IFSTTAR, 38000 Grenoble, France; 5 Landesamt für Geologie und Bergbau, Mainz, Germany; 6 TU Berlin, Institute of Applied Geosciences, Berlin, Deutschland Germany

**Keywords:** Geophysics, Volcanology

## Abstract

Continuous monitoring of volcanic gas emissions is crucial for understanding volcanic activity and potential eruptions. However, emissions of volcanic gases underwater are infrequently studied or quantified. This study explores the potential of Distributed Acoustic Sensing (DAS) technology to monitor underwater volcanic degassing. DAS converts fiber-optic cables into high-resolution vibration recording arrays, providing measurements at unprecedented spatio-temporal resolution. We conducted an experiment at Laacher See volcano in Germany, immersing a fiber-optic cable in the lake and interrogating it with a DAS system. We detected and analyzed numerous acoustic signals that we associated with bubble emissions in different lake areas. Three types of text-book bubbles exhibiting characteristic waveforms are all found from our detections, indicating different nucleation processes and bubble sizes. Using clustering algorithms, we classified bubble events into four distinct clusters based on their temporal and spectral characteristics. The temporal distribution of the events provided insights into the evolution of gas seepage patterns. This technology has the potential to revolutionize underwater degassing monitoring and provide valuable information for studying volcanic processes and estimating gas emissions. Furthermore, DAS can be applied to other applications, such as monitoring underwater carbon capture and storage operations or methane leaks associated with climate change.

## Introduction

At most volcano observatories, continuous seismicity and ground deformation are well monitored through ground-based sensors (e.g.,^1^) and remote sensing approaches (e.g.,^2^). However, besides the mechanical behavior, the continuous volcanic gas release is another fundamental process to investigate (e.g.,^3^). Volcanic gases are a main trigger of explosive eruptions. Still, while highly relevant for interpreting volcanic unrest’s evolution (e.g.,^4^), volcanic gases are generally only periodically determined from discrete direct sampling surveys (e.g.,^5^) or through satellite images (e.g.,^6^). Furthermore, most measurements are performed on known gas plumes, but gas can still escape diffusely through crustal faults^7^, soil or water bodies^8,9^, even during quiescence^8^. At water-surface interfaces, $\hbox {CO}_{2}$ is generally measured using floating accumulation chambers^10^. Extensive research has been completed to estimate $\hbox {CO}_{2}$ emission from volcanic lakes^11^ and monitor them through time (e.g.,^12,13^). Yet, $\hbox {CO}_{2}$ can dissolve in the water column when ascending towards the surface (e.g.,^14^) and is therefore not captured by this method; the exact degassing locations remain unknown. By lacking techniques sensitive to underwater degassing, we can therefore miss substantial hazards^15^ but also potentially limit our understanding of volcanic processes.

The presence of a body of water with underwater degassing provides an underexploited opportunity to investigate volcanic degassing through non-conventional means. Bubbles are easily observed in volcanic lakes^14^ and submarine volcanic environments^16^. They can either nucleate^17^ or seep through lake/sea bottom as already formed bubbles. Bubbles then rise through the water column and can dissolve or reach the surface^14^. External drivers such as lake level or tides^18^ can change and influence the level of bubbling observed at the surface. Contrary to subaerial settings, the acoustic detection of gas is straightforward in aquatic environments^14,16,19^. This is because nucleating, oscillating, and collapsing gas bubbles are powerful acoustic sources allowing hydrophones to track gas fluxes at high temporal resolution before mixing and dilution in the atmosphere^17,20^, similar to hydrocarbon gas plume monitoring^21,22^. Yet, hydrophones provide single-point measurements, and acoustic waves quickly attenuate within a distance of a few meters to the source^16,23^, limiting their monitoring capabilities. Therefore, models rely on wide interpolation. This also limits the reliability of $\hbox {CO}_{2}$ output estimates from volcanoes^24^.

An emerging technology called Distributed Acoustic Sensing (DAS) has the potential to provide acoustic measurements at an unprecedented spatio-temporal resolution by turning fiber-optic (FO) cables into high-resolution vibration recording arrays^25^. An interrogator unit sends a series of coherent laser pulses into an FO cable and measures the back-scattered photons over successive fiber segments (i.e., the gauge lengths). When the fiber is distorted due to vibration, the resulting phase shift of optical backscatters is proportional to the changes in path length over the gauge lengths^26^. DAS technology can effectively record continuous vibrations with thousands of single-component channels. It is capable of real-time data analysis for a virtually unlimited deployment duration as long as the interrogator unit is connected to a power source. While DAS has been extensively used in geosciences (e.g.,^27,28^) and industry (e.g.,^29,30^), its underwater and volcanology monitoring applications are still in their infancy (e.g.,^31–33^).

**Figure 1:**
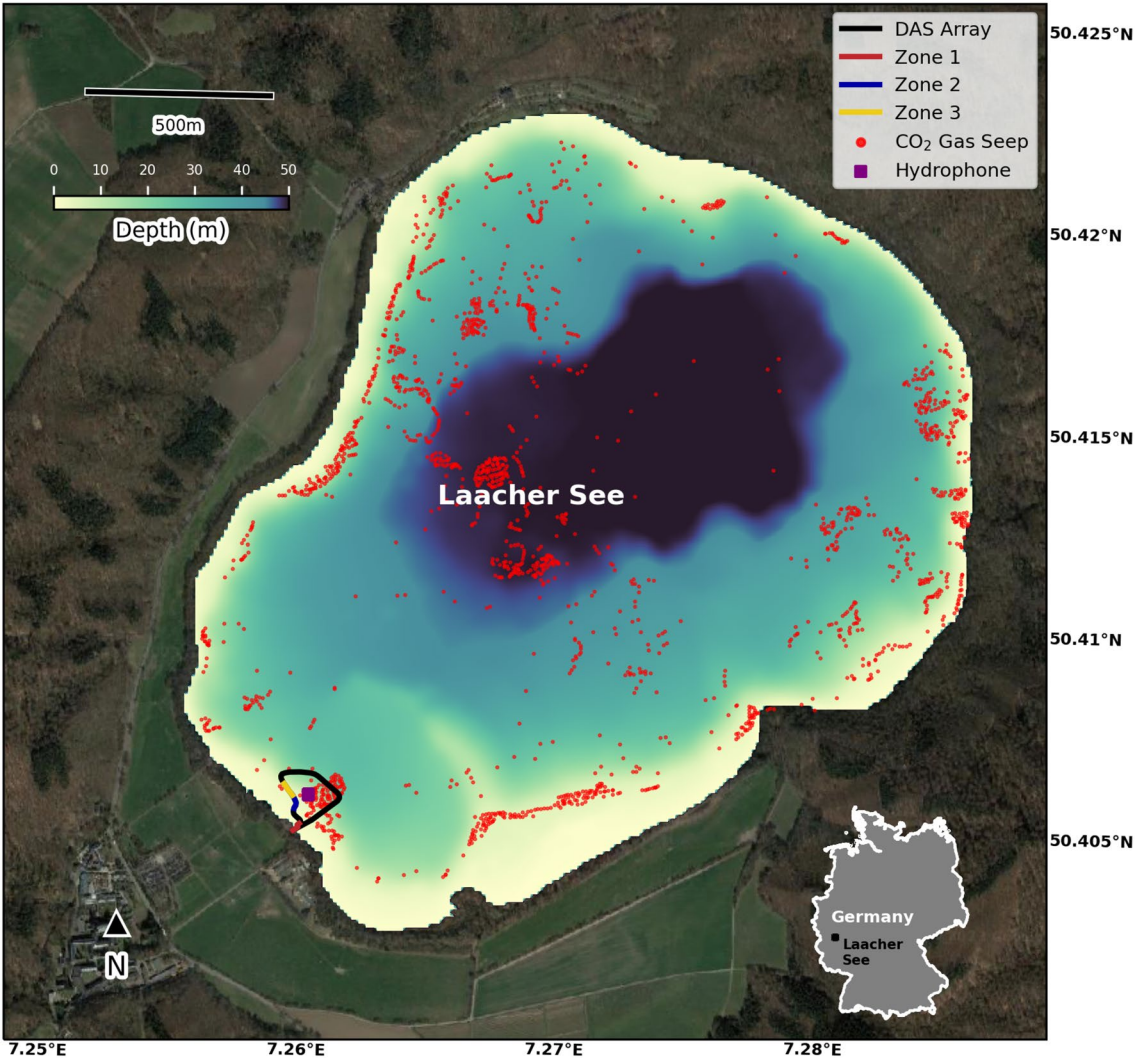
Aerial view of the Laacher See Volcano. Map of the LSV and the fiber-optic array (continuous line). The subsections of the cable highlighted in red, blue, and yellow correspond to three zones with enhanced gas emissions (Zone 1: channels 130–170, Zone 2: channels 220–260, and Zone 3: channels 260–300; same colors as Fig. 4). The purple square represents the hydrophone location inside the fiber optic loop. Each red dot depicts a $\hbox {CO}_{2}$ gas seep identified by Albers et al.^38^. The high-resolution bathymetric map is from Albers et al.^38^. The map is generated with Cartopy v0.22.0^40^.

Here, we show that DAS technology has the potential to improve the understanding of underwater degassing processes. We interrogated an underwater fiber-optic cable at Laacher See volcano (LSV), in the Eifel region, Germany (Fig. 1). The 2-km wide LSV caldera lake formed $\sim$13000 years ago^34^ during a major Plinian eruption that released a magma volume of 6.7 km^3^ (VEI 6), making it one of the largest documented Quaternary eruptions in central Europe (e.g.,^35^). The volcanic area has been experiencing seismic unrest and ground deformation signaling an influx of magmatic fluids^36,37^, and low-intensity gas seepages have been extensively mapped^38^. A large seismological experiment is currently exploring the structure and activity of the magmatic system^39^. Our DAS experiment allowed us to detect and analyze plentiful bubble acoustic signals in various lake areas. This contribution demonstrates the sensitivity of fiber-optic cables to underwater degassing events and its potential for monitoring underwater volcanic emissions.

## Results and discussion

### Data collection

We immersed a 500-m fiber-optic cable in the southeast region of the lake where $\hbox {CO}_{2}$ gas seeps were previously identified (^38,41^; Fig. 1). The cable is a 3.2 mm diameter double-coated tactical armored cable with a central metal tube containing one single optical fiber strand. It has a net weight of 10.5 kg/km, meaning its density is slightly higher than freshwater, thus it naturally sinks at the lake bottom. Professional divers set the cable at maximum depths of $\sim$25 meters under a few centimeters of alluvial sediments to achieve a better ground coupling. An optical damper was installed at the end of the cable to decrease unwanted laser reflections. The georeferencing of the cable is described in the “Methods section”.

The fiber was then interrogated with a Silixa iDAS interrogator installed in a near-shore fisherman’s house. Raw data were saved in the form of phase change (proportional to strain rate) at 5000 and 8000 Hz for a duration of 18 and 22 hours, respectively. Data were collected at a 1-m spatial sampling with a 10-m gauge length with a total of 640 recording channels. The first 120 channels are inside the DAS interrogator. Channels 120-130 are inland and the last 60 channels are near the lake edge with uncertain coupling conditions and extensive optical noise (Fig. S5). We use the remaining 450 channels situated underwater with more stable coupling conditions and acting as hydroacoustic sensors for subsequent analysis.

In addition, two hydrophones were installed at the center of the loop (Fig. 1). The microphones (SNAP from Loggerhead company) recorded the ambient acoustic noise at 44.1 kHz. The gain level was set to the minimum based on the noise level recorded during previous experiments^41^.

**Figure 2:**
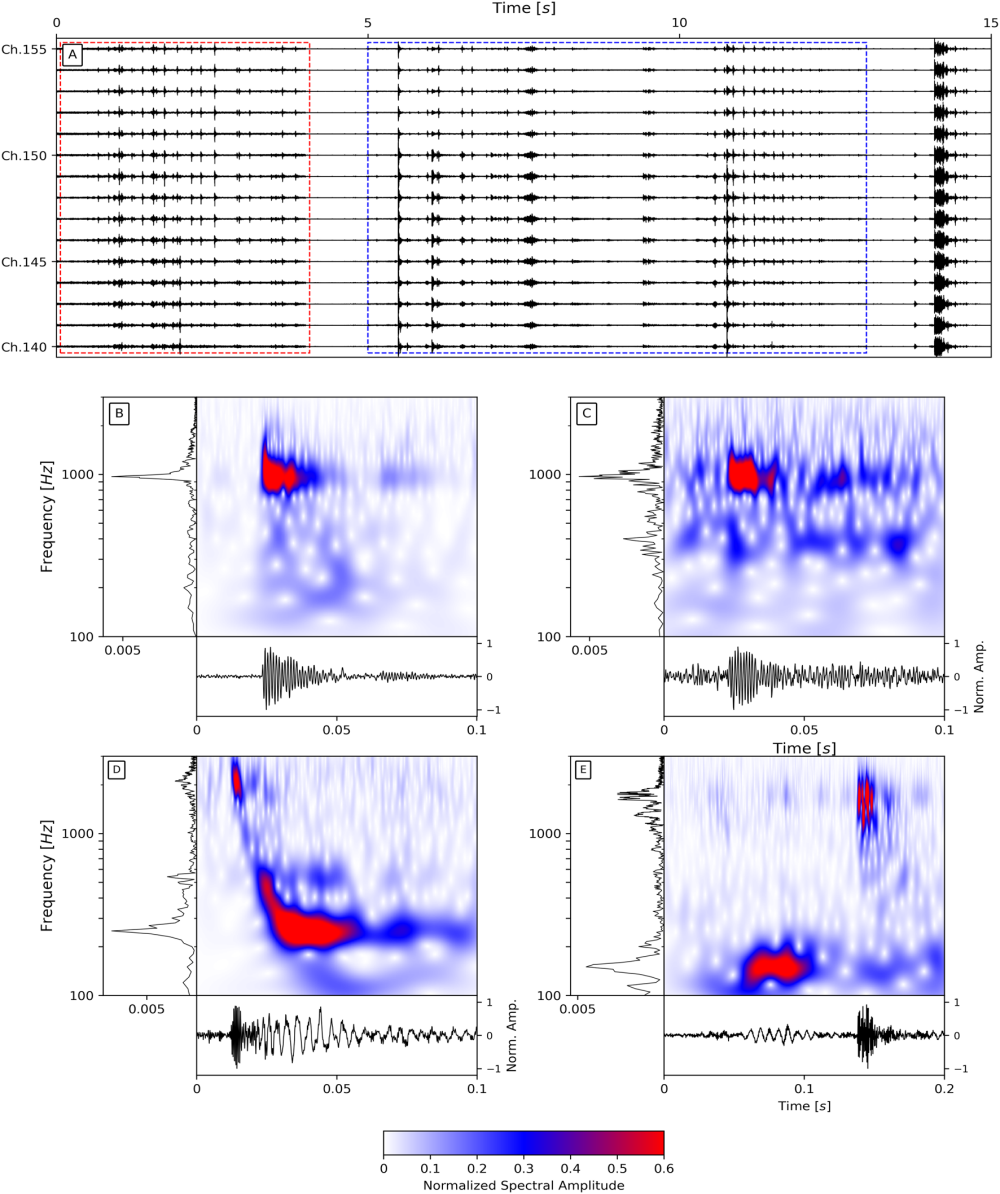
Examples of bubble events. Frequency content, spectrogram with wavelet transform (using Morlet wavelet), and waveform of a bubble are shown in the top left, top right, and bottom subpanels of subplots B-E, respectively. (**A**) 15-meter record section (channels 140-155, which is a subsection of Zone 1) over a 15-s period recorded with DAS. The amplitudes of the signals in the red and blue boxes are multiplied by a factor of 10 and 5, respectively. (**B**) Typical monochromatic Type 1 bubble acoustic signal recorded with DAS. (**C**) Typical monochromatic Type 1 bubble acoustic signal recorded with a hydrophone. (**D**, **E**) Typical bimodal bubble acoustic signals (Type 2 and Type 3 respectively) recorded with DAS.

### Bubble hydroacoustic signals

Visual inspection of the records revealed the presence of countless impulsive events continuously occurring throughout the recording period (Fig. 2A). Both DAS and hydrophone data show that these acoustic signals (hereafter referred to as “bubble events”) hold the characteristic signatures of bubbles rising to the surface^17,42^, which was also confirmed with videographic data recorded in near-shore sections of the cable (Movie S1).

Over the wealth of waveforms observed, three main types of bubble events stand out (Fig. 2B,D,E). Figure 2B,C (DAS vs. hydrophone) show a typical oscillating lightly-damped bubble acoustic signal (Type 1 bubble). It reaches its maximum amplitude in the first milliseconds and then quickly decays as a classical damped oscillator^43^. These bubble-acoustic signals reveal one dominant frequency peak, generally between $\sim$100 and $\sim$2000 Hz. The largest and clearest events have a duration of up to $\sim$100 ms, but the vast majority have a duration of $\sim$30 ms. Another type of bubble events often start with a high-frequency impulsive onset ($\sim$kHz) followed by lower frequency oscillations ($\sim$100-1000 Hz), and have a duration ranging between 50 ms up to 200 ms (Type 2 bubble, Fig. 2D). The other but less frequent type of bubble events start with a low-frequency onset under $\sim$50 Hz, followed by a high-frequency wave packet between $\sim$100 and $\sim$2000 Hz (Type 3 bubble, Fig. 2E). This type of bubble events tends to have a longer duration over $\sim$100 ms.

These text-book bubble events^44^ reflect three distinct bubble nucleation processes^16,17,42^. The monochromatic Type 1 bubble event characterizes a bubble detaching from a rigid surface, such as that of an orifice in a well-formed sediment bed, but without extended interactions with this surface. The bimodal Type 2 bubble events indicate an interaction with the sediment bed^42^. In Fig. 2D, the high-frequency onset of a Type 2 bubble is generated by a void space opening and subsequent sediment motion. Then, the lower-frequency signal is caused by free oscillations of the newly formed bubble, right after it detaches from the sediments. In Fig. 2E, the very low-frequency oscillation of a Type 3 bubble may reflect a turbulent interaction of the mixture comprising water, dissolved volatiles, and nucleated gas bubbles with conduits in the sediments before the bubble escapes in the water column^17^.

In all cases, the bubble waveforms hold both the signature of their interactions with the sediment bed during or after nucleation^17^, and an indication of their sizes^43^. The shallow sediments at Laacher See are weakly cohesive^45^. Therefore, the particles at the surface can easily be displaced along with the gas motion over short time scales. Thus, even at a constant gas throughput, an orifice can rapidly change its shape, giving rise to a myriad of processes and, consequently, bubble waveform onsets over time. Once the bubble is formed and oscillates in the water column, its dominant frequency is proportional to its size^44^. Therefore, following Minnaert^43^ equation (Eq. 1 in “Methods”), it is possible to estimate the volumes of gas associated with a given bubble event.

**Figure 3:**
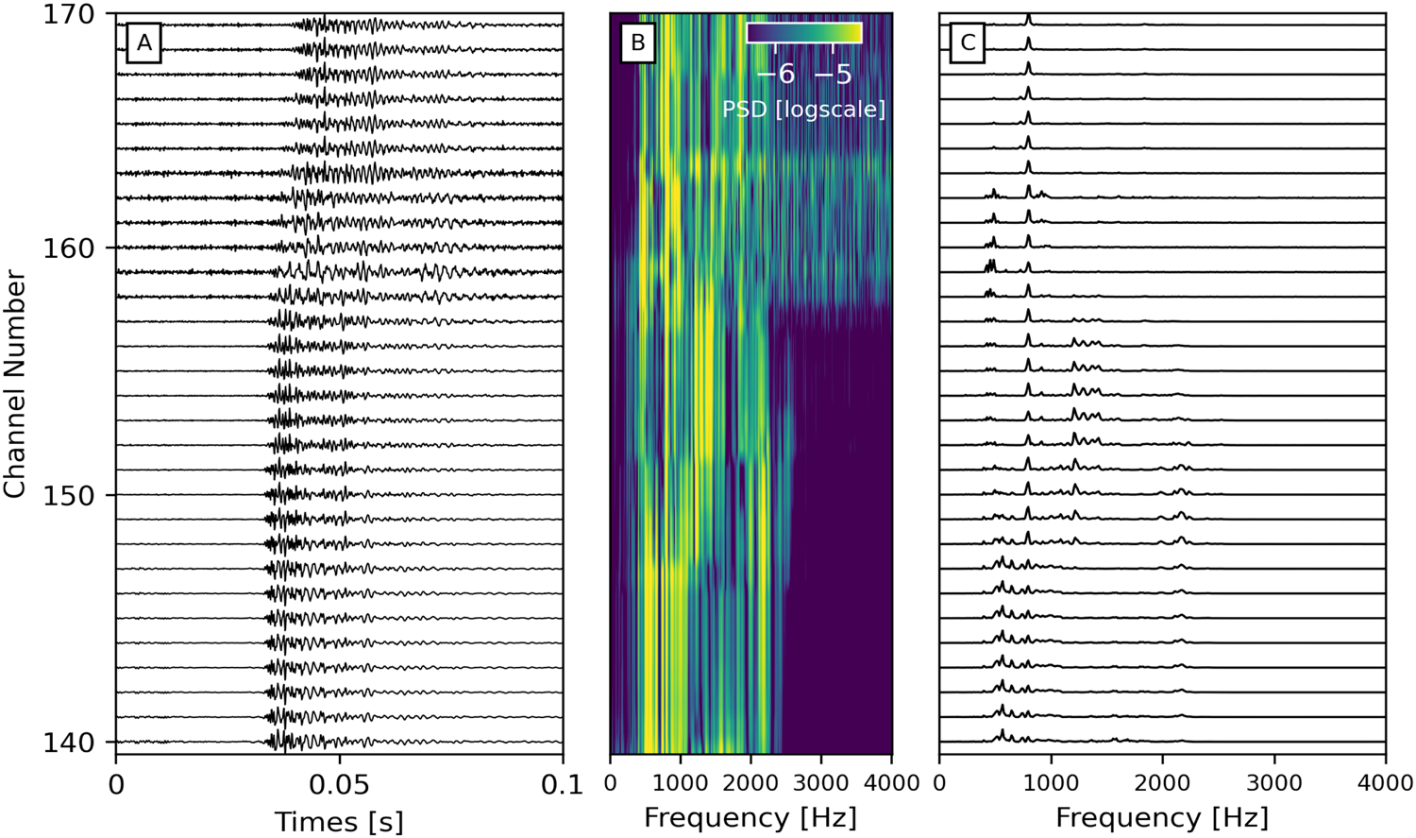
Evolution of a bubble waveform along a section of the array. (**A**) Bubble waveforms between channels 140-170 (i.e., over 30 m). (**B**) Normalized Power Spectral Density (PSD) functions of the same bubble shown in A). Brighter and darker colors correspond to higher and lower energy, respectively (in log scale). (**C**) Spectra of the waveforms shown in (**A**). We observe that the higher frequency peak around 2200 Hz is not recorded at all channels, likely due to attenuation effects.

Overall, bubble events close to each other exhibit similar waveforms and spectra. Yet, they can significantly change along the channels and rapidly attenuate. A given bubble event depicts a slightly different waveform based on where it is recorded on the sediment bed (Fig. 3). Indeed, both increasing and decreasing frequencies are recorded with increasing distance from the bubble seep, but the onset high-frequency signal is often lost moving away from the seeps (e.g., Figs. 3 and S1−S5). This likely relates to bubble attenuation, different coupling conditions, and/or local site effects. As a result, we could only identify the same bubble signals over a 30-channel section with similar waveforms. In addition, a smearing effect is caused by our gauge length of 10 meters. Yet, unlike hydrophones (e.g.,^20^), DAS provides spatial resolution and indications of the interactions with the lake bed, thereby delivering a more complete picture of gas-sediment interactions and degassing processes.

### Spatiotemporal analysis of bubble events

**Figure 4:**
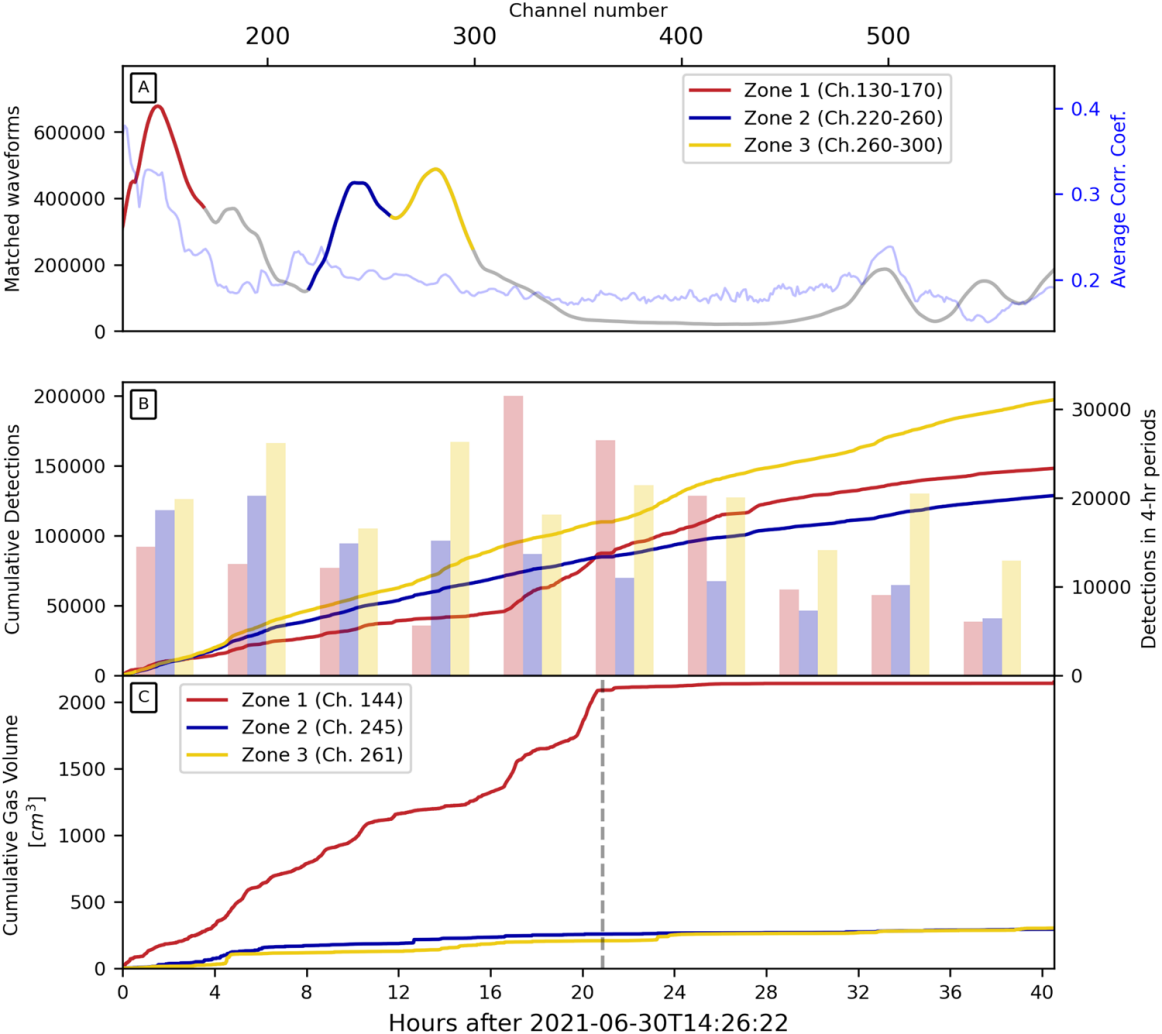
Number of bubble events recorded. (**A**) Number of matched bubble waveforms and average cross-correlation coefficients of the matchings of each channel between channels 130-580. The three high-detection zones (i.e., zones 1, 2, and 3) are colored red, blue, and yellow, respectively. (**B**) The total number of WSS detected bubble events in different high-detection zones. (**C**) Cumulative gas seepage volume of the three zones. The colors in (**B**) and (**C**) correspond to the color code in (**A**). The black dashed vertical line in (**C**) represents the Cluster 1 stoppage in Fig. 5.

Based on an initial population of thirty manually extracted waveforms (e.g., Fig. 2), we applied a waveform similarity search, followed by a channel-to-channel template matching over the entire length of the immersed fiber array (see Method section). We performed the detection analysis on the 450 underwater channels (channels 130–580). The bubble event search allowed us to identify three different main zones with active seepage along the cable (Zone 1: channels 130-170, Zone 2: channels 220-260, and Zone 3: channels 260-300 in Figs. 1 and 4A). The total number of bubble events detected after association exceeds 470000 from three zones aggregated over a recording period of 40 hours (Fig. 4B). On average, the rate at which bubble events were detected is about one bubble per second, which is in accordance with some of the videographic data (Movie S1) collected near the shore. We then estimated the temporal trend of gas emission volume for further calculations (see *Methods* section). Fig. 4C shows the evolution of gas seepage volume from three zones. Knowing that three zones have comparable numbers of bubble events (Fig. 4B), the significantly higher estimation of gas volume in Zone 1 indicates a greater proportion of low-frequency events in this area.

**Figure 5:**
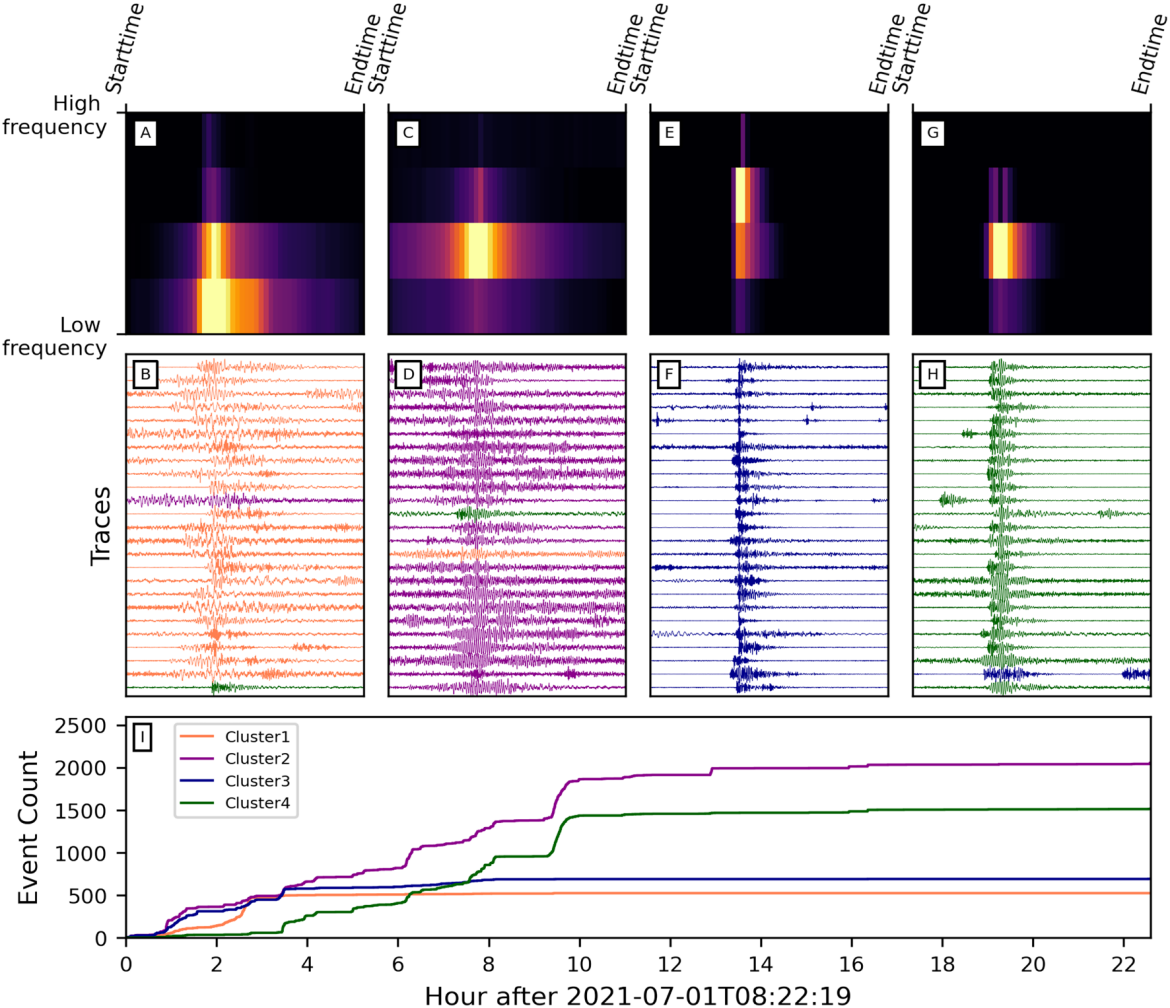
Clustering result of bubble events from a single channel (channel 144). (**A**) Centroïd bottleneck matrix of K-means Cluster 1, which is characterized by dominant low-frequency energy. (**B**) Waveform examples of bubble events from cluster 1. Color represents the predicted cluster labels from our clustering algorithm. Coral, dark magenta, dark blue, and dark green represent Cluster 1, Cluster 2, Cluster 3, and Cluster 4, respectively. Color mismatches represent inconsistencies between manual labels and predicted labels. (**C**, **D**) Same as (**A**, **B**), but for cluster 2, which has consistent middle-frequency energy. (**E**, **F**) Same as (**A**, **B**), but for cluster 3, which features significant high-frequency energy and simultaneous weaker middle-frequency energy. (**G**, **H**) Same as (**A**, **B**), but for cluster 4, which is characterized by weak high-frequency energy followed by stronger middle-frequency energy. (**I**) Cumulative event count over time of four clusters. The same set of colors is used as in (**B**, **D**, **F**, **H**). Note that a different model is used than the model for (**A**−**H**).

Understanding the time evolution within the bubble groups can help better constrain gas seepage patterns. We implemented a clustering algorithm for high-SNR bubble events based on the temporal-spectral characteristics of bubbles. It consists of a Convolutional Neural Network (CNN) for dimensionality reduction of spectrograms and a K-means cluster estimator (see Method section). Due to the different input dimensions resulting from signals with different sampling rates, it becomes challenging to train mixed-frequency signals using a uniform CNN scheme. In this analysis, we focused on bubble events sampled at 8000 Hz. We applied such analysis to channel 144 from Zone 1, which has the most high-SNR detections (4784 events). By both manually scrutinizing the bubble waveforms and carrying out elbow tests on the K-means clustering algorithm (Fig. S6), we defined four clusters for the dataset. Training with only the first 1000 bubbles, the algorithm yielded an accuracy of 95.5%. Fig. 5A-H shows centroid matrices and multiple events for each cluster. While we show three commonly-recognized bubbles in Fig. 2, such events are archetypal examples showing features comparable with other studies (e.g.,^17,42,45^). However, the four groups from the clustering algorithm do not have the exact same features and sometimes show hybrid bubble characteristics (i.e., low and high frequencies) from different groups, likely due to more complex sediment/water interaction with bubbles. Nevertheless, these four clusters exhibit distinctly observable characteristics both in the temporal and spectral domains. Cluster 1 features more low-frequency ($\lesssim$ 600 Hz) energy. Events in Cluster 2 have temporally stable middle-frequency ($\sim$ 600–1600 Hz) energy. The impulsive arrival and higher frequency ($\gtrsim$ 1600 Hz) component characterize cluster 3. Cluster 4 has a strong middle-frequency component following the arrival of high-frequency energy. We then expanded the analysis to the complete set of events. Fig. 5I shows the temporal distribution of the events. We observe the interruption of Cluster 1 and Cluster 2 at around 3 hours after start time. The occurrence rates of Cluster 3 and Cluster 4 also dramatically decreased after 10 hours. Low-frequency Cluster 1 signals have a much larger bubble radius and contribute a significant amount of gas seepage volume. The stoppage of Cluster 1 signal after 3 hours corresponds to the Zone 1 gas volume pattern well (black dashed line in Fig. 4C). When including all events, retraining the model, and optimizing the parameters, the best accuracy reaches 77%. Compared with the model using the first 1000 events, we attribute this performance degradation to the uneven distribution of bubble groups across time and waveform temporal variations within bubble clusters. Changes in physical processes such as pressure, temperature, and soil conditions may cause such waveform variations.

### Bubble characteristics at Laacher See

We do not know the compositions of the bubbles during our field experiment at these locations, but existing studies have dominantly found magmatic $\hbox {CO}_{2}$ in bubbles^38,46^. Due to their similarity with bubbles studied elsewhere using hydrophones (e.g.,^17,42,47^), we ascribe these signals to $\hbox {CO}_{2}$ bubbles.

We now compare our results with bubble events recorded by a single hydrophone at Yellowstone Lake (USA) and during laboratory experiments. Contrary to the hybrid frequencies-bubble signals recorded by hydrophones in Yellowstone Lake^17^, the onset of the low-frequency (100-1000 Hz) signals at Laacher See only starts when the high-frequency amplitude vanishes (Fig. 2C). High-frequency onset and overall signals at Laacher See also last longer (an order of magnitude higher compared to Yellowstone). Finally, we note the absence of very low frequencies (2-40 Hz) associated with the high-frequency onsets, except for the largest bubbles (Fig. S7). Laboratory experiments^42^ instead show similar waveforms to Laacher See with high-frequency signals followed by low-frequency oscillations only starting when the bubbles are detached from the sediments.

Taken altogether, these observations suggest that bubbles are generally not being nucleated beneath the fiber optic cable at Laacher See but seep through the watery sediments as already-formed $\hbox {CO}_{2}$ gas bubbles rather than dissolved $\hbox {CO}_{2}$. Bubble nucleation events typically start with an impulsive, high-frequency onset generated by the opening of void space in the liquid, and the subsequent, lower-frequency signal is generated by free oscillations of the newly formed bubble^17,48^. Yet, we note a single exception at Laacher See corresponding to the largest amplitude bubble signals having very low-frequency oscillations associated with the high-frequency onsets, as observed in Yellowstone (Fig. S7). Such a signal possibly bears the characteristics of bubble nucleation within the sediments.

### The potential of DAS for hydroacoustic monitoring on active volcanoes

Optical fiber seismic hydrophones have recently been successfully designed to detect earthquakes (below 100 Hz)^49^. Our study indicates that fiber-optic cables interrogated by the DAS technology are sensitive to gas bubbles that are tiny acoustic events also sensed by single hydrophones. DAS technology, however, provides numerous sensors with spatial resolution down to 1 meter. Compared to hydrophones, fiber-optic cables therefore not only allow us to characterize degassing but also study its spatial evolution. Consequently, this technology opens new perspectives to study attenuation in three dimensions and the possible influence of the water column on wave propagation. It also provided detailed information on ground vibrations during the nucleation and seepage processes. Being coupled with the ground, we anticipate critical improvement in our understanding of gas-sediment interactions, such as gas migration pathways or nucleation.

In addition, some fiber-optic cables can be operated in various environments: at large depths with high pressures, high temperatures, and even in acidic environments^50–52^. They would also open new ways to monitor underwater volcanoes as the cables can be left permanently without much maintenance required. In addition, the interrogation can be carried out inland. Yet, we found that the coupling appears critical. At underwater calderas or craters, such as Santorini (Greece) or Hunga Tonga-Hunga (Tonga), this would provide complementary monitoring observations (e.g.,^53^), such as gas volume over time, but also improved estimates of global volcanic $\hbox {CO}_{2}$ emissions^24^. Overall, DAS technology holds great promise to monitor underwater volcano degassing at high resolution; 70% of volcanoes are found underwater. But this also opens the door to monitoring underwater $\hbox {CO}_{2}$ sequestration operations or $\hbox {CH}_{4}$ leaks associated with permafrost or hydrate destabilization.

## Materials and methods

### Cable geolocalization

**Figure 6:**
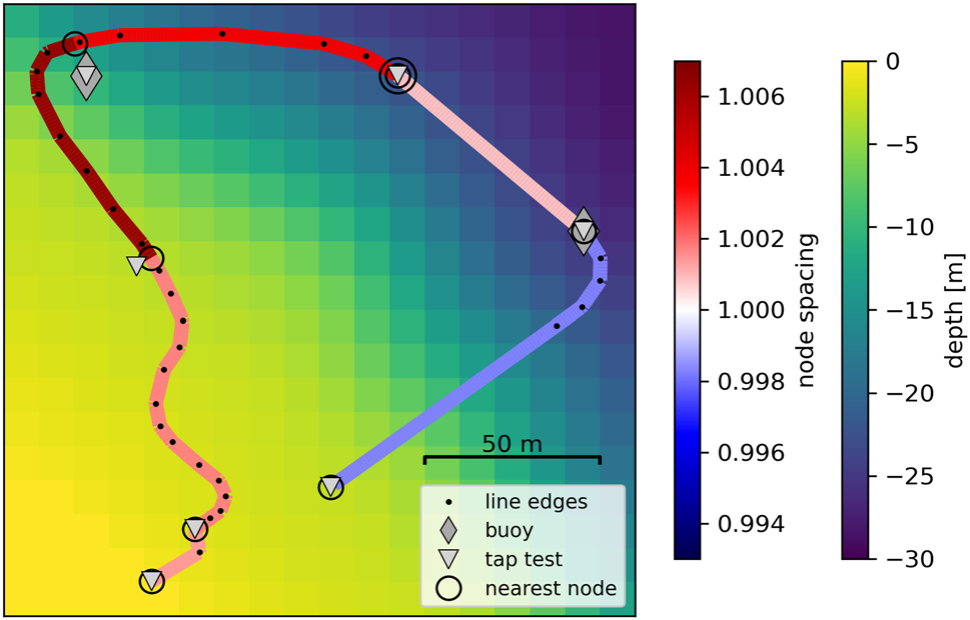
Fiber-optic array geometry. The segmented line delineates the fiber trajectory where the color of a segment between two tap tests corresponds to the inter-node spacing (blue to red color scale). Triangles and circles mark tap-test locations and their corresponding nearest sensors, respectively. The sensor marked with two nested circles indicates the location of the tap test whose recording is shown in Fig. 7. The underlying colormap depicts the bathymetry of Laacher See near the fiber.

Geophysical measurements with DAS require knowing the geographic trajectory of the fiber and the distribution of the virtual sensors (each centered at their respective gauge length). If the fiber trajectory is known and accessible, the task of georeferencing may be accomplished by simply tapping the fiber (tap-test) and associating the stimulated channel with the respective geolocation of the tap. In this experiment, the fiber is submerged in up to 25 m of water (Fig. 6) and, therefore, is not directly accessible.

We originally designed the fiber array as an equilateral triangle. To do so, we used two buoys and the fisherman’s house to mark the corners of a triangle with a perimeter almost equal to the fiber’s length (e.g., 500 m). While rolling out the fiber from the boat traveling from one buoy to another, maintaining a straight course was not always possible due to obstacles near the shore and the drift induced by strong winds. The fiber was also laid around each buoy in a wider radius to prevent sharp bends and possible damage.

**Figure 7:**
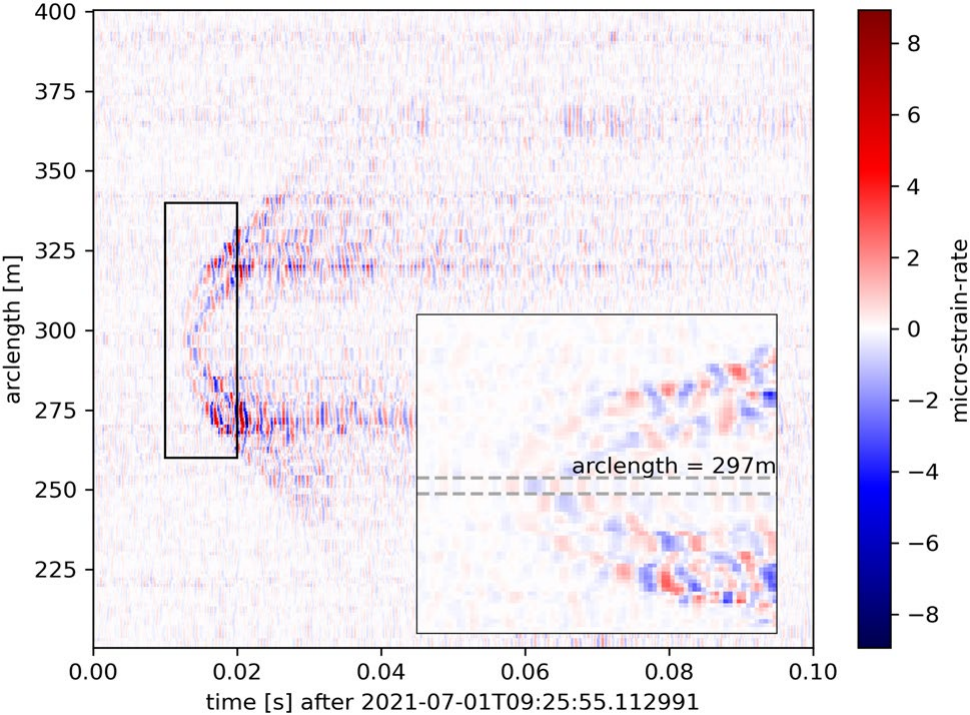
DAS-recording of a tap on the boat’s hull. The inset enlarges the area marked by the rectangle. The horizontal dashed lines depict the DAS channel that records the tap signal first. The tap location is marked in Fig. 6.

After divers coupled the fiber on the lake floor, we performed tap tests by hitting the boat’s hull at six locations, i.e., at the buoys and halfway between them (Fig. 6). We then picked the channels recording the earliest arrival of the tap event (Fig. 7) and associated it with the respective tap-test location. Locations of the channels in between were preliminarily obtained by equidistantly distributing them along the straight connecting the respective tap locations.

The uncertainty of the obtained sensor locations can be attributed to several sources. First of all, the GPS locations were retrieved with an ordinary smartphone yielding an accuracy down to several meters only. The taps were not timed, so their travel time to the lake floor could not be directly assessed. Thus, we assumed that the picked channels are the nearest to the tap location, but they were not directly associated with it. Due to the bathymetry, the nearest sensors may not lie vertically below the boat.

After the initial realization of this procedure, we observed a misfit between the optical and the mapped fiber length. Between two picked channels, the optical and mapped fiber lengths are given by the number of channels multiplied with the spatial sampling period and the arclength of the fiber-optic cable trajectory on the lake floor between the corresponding tap locations, respectively. The optical fiber length between picked channels resulted longer than the mapped trajectory between tap locations on all fiber segments. This is equivalent to mapping the channel locations at an inter-channel distance that is smaller than the chosen spatial sampling period (red-blue colorbar, Fig. 6). We thus manipulated the fiber trajectory manually to the best of our knowledge, accounting for the previously described detours during the laying out, thus effectively increasing the mapped fiber length until it matched the optical length (i.e. mapped inter-channel distance equals the chosen spatial sampling period, Fig. 6). However, for fiber segments containing curves there is a multitude of possible trajectories satisfying this condition thus increasing the uncertainty of channel locations on such.

Several measures could improve and facilitate future deployments in a lake. Projecting the geo-spatial trace of the divers and fixing the fiber on the lake floor would probably yield the most accurate trajectory. If the fiber lies at depths unreachable by divers, spatially dense tap tests should be accurately timed and located with triggered GPS clocks and differential GPS locations, respectively. The travel times of the induced acoustic waves could then be accurately picked, and their inversion could yield reasonably good virtual sensor locations. Systematic variation of the retrieved fiber trajectory could then yield an ultimate assessment of the trajectory’s uncertainty. Each alternative trajectory must meet the condition that its mapped arclength and the optical distance remain numerically equal (between tap-test locations and the corresponding pair of picked channels).

### Bubble detection

We designed a template-matching (TM) procedure to detect bubbles in an automated way along the fiber. TM consists of using previously detected events (i.e., the templates) to scan the continuous data by performing cross-correlations to find new events (^54,55^, i.e., the detections). Because our dataset consists of data with different sampling rates, the detection was conducted separately for 5000 Hz and 8000 Hz data with the same procedure. We first ran a preliminary Waveform Similarity Search (WSS) over all channels to create a template database, by utilizing 60 manually-selected high-SNR bubble waveforms and computing cross-correlation with the continuous time series. We used a common Median Absolute Deviation (MAD) threshold of 9 to identify similar waveforms^54,56^. Fig. 4A summarizes the WSS results, highlighting three 40-meter zones of higher seepage activity along the cable (i.e., channels 130-170, 220-260, and 260-300). In total, WSS matched 112.32 million waveforms from the 640 channels combined. Yet, in our processing workflow, a given bubble event can be matched by several templates over multiple channels. Hence, most events are counted multiple times. Consequently, the large number of matched waveforms needed to be further constrained. Thus, we examined the WSS detection results and only kept events with high SNR and spatial consistency (e.g., observed over a minimum of 20 channels) as templates. In total, we kept 5210, 893, and 1573 templates for three zones, respectively. We used the same $MAD=9$ threshold for TM. Considering the relatively large amount of templates for the first section, we ran TM on Zone 1 by small batches of templates, demonstrating the relative completeness of our detection (Fig. S8). TM results are shown in Fig. 4B. From the 40-hour experiment period, 148341, 128710, and 197397 events are found for the three zones by TM, respectively.

### Gas throughput estimation

Using the dominant frequency, we can estimate bubble sizes assuming they are spherical. The diameters of these bubbles are on the order of 0.5 to 1.6 mm using Minnaert^43^’s equation:

1 $$\begin{aligned} r = \frac{(3 P\gamma /\rho )^{\frac{1}{2}}}{2 \pi f}; \end{aligned}$$

where P is the hydrostatic pressure (1082 kPa), $\gamma$ is the ratio of specific heats of the gas in the bubble (1.3 for CO2), $\rho$ is the density of the liquid (1000 kg/$m^3$), and *f* is the dominant frequency. Estimated sizes are compatible with bubble sizes estimated using independent active and passive hydroacoustic instruments at nearby lake locations (e.g., 20 meters away), where we could also visually track bubbles rising towards the surface (Movie S1). The volume for each bubble ranges between 0.002 and 0.007 $m^3$.

### Bubble clustering

A bubble can be characterized based on its dominant frequencies and arrival time move-outs of different frequency groups (Fig. 2). Therefore, we selected high-SNR events and computed the wavelet transform of those events to retrieve the temporal-spectral information. We applied such analysis on the single channel with the most detections (i.e., channel 144; where we had video of a bubble rising towards the surface (Movie S1)), where 4784 bubble events pass the SNR threshold. We shifted and trimmed the bubble recordings so that each trace covers 0.2 seconds and all bubbles were well aligned. Wavelet transform converts the time-series of bubbles into $128 \times 1600$ 2D matrices (128 spectral estimates, 1600 temporal estimates).

We first fed raw 2D matrices from wavelet transform to the K-means clustering algorithm. By doing an elbow test, we decided to cluster the bubbles into four groups (Fig. S6). We then manually labeled 400 events for clustering accuracy estimation. This K-means cluster separator serves as a baseline model and yields an accuracy of 62.5%. Mousavi et al.^57^ designed an unsupervised learning algorithm for clustering teleseismic events and local earthquakes. It consists of a set of convolutional layers and a K-means clustering layer, integrating the loss from both components. To improve the clustering performance, we adopted a similar CNN structure to reduce the dimensionality of the raw inputs and concentrate the temporal-spectral information to $4 \times 50$ size matrices. We selected four groups for subsequent K-means analysis. After training the model for multiple iterations for the matrix reconstruction loss to converge, we used bottleneck layers to feed the K-means clustering algorithm. We first tried such a CNN+K-means model with training with the first 1000 bubble events. We manually labeled 400 bubbles for clustering accuracy estimation, with 200 of them from the first 1000 bubbles and the other 200 from the rest of the bubbles. Accuracy is measured as the percentage of correctly labeled events of all clustered events. By doing so, 200 events from the first 1000 bubbles (serving as a training dataset) yielded an accuracy of 95.5% while the other 200 events (outside the training dataset, serving as a test group) returned a lower accuracy of 75%. These significant accuracy improvements to the K-means-only model demonstrate the efficacy of the convolutional network for dimension reduction. In addition, such results also suggest a preference for the complete inclusion of bubble events into the model training set, providing access to large-memo computational resources. Therefore, we repeated the same procedure with the inclusion of all bubbles. Knowing the stochastic nature of the convolutional network and data shuffling, we repetitively trained the model on a GPU card 100 times and evaluated the performance with labeled data. The best-performance model is then adopted in the main text and shown in Fig. 5.

### Supplementary Information

Supplementary Information.

Supplementary Movie S1.

## Data Availability

All data needed to evaluate the conclusions in the paper are present in the paper and/or the Supplementary Materials. The data and codes used to reproduce the paper’s figures will be made publicly available after review and before the eventual acceptance of the manuscript.
