# Author Correction: Monitoring underwater volcano degassing using fiber‑optic sensing

**DOI:** 10.1038/s41598-024-57577-y

**Published:** 2024-03-26

**Authors:** Corentin Caudron, Yaolin Miao, Zack J. Spica, Christopher Wollin, Christian Haberland, Philippe Jousset, Alexander Yates, Jean Vandemeulebrouck, Bernd Schmidt, Charlotte Krawczyk, Torsten Dahm

**Affiliations:** 1 Laboratoire G-Time, Department of Geosciences, Environment, and Society, Université Libre de Bruxelles, Brussels, Belgium; 2 Department of Earth and Environmental Sciences, University of Michigan, Ann Arbor, MI USA; 3 GFZ German Research Centre for Geosciences, Potsdam, Deutschland Germany; 4 ISTerre, Université Grenoble Alpes, Université Savoie Mont Blanc, CNRS, IRD, IFSTTAR, 38000 Grenoble, France; 5 Landesamt für Geologie und Bergbau, Mainz, Germany; 6 TU Berlin, Institute of Applied Geosciences, Berlin, Deutschland Germany

Correction to: *Scientific Reports* 10.1038/s41598-024-53444-y, published online 07 February 2024

The original version of this Article contained an error in Reference 38, which was incorrectly given as:

“38. Goepel, A., Lonschinski, M., Viereck, L., Büchel, G. & Kukowski, N. Volcano-tectonic structures and co2-degassing patterns in the Laacher see basin, Germany. *Int. J. Earth Sci.* **104**(5), 1483–1495 (2015).”

The correct reference is listed below:

“38. Albers, S., Vandorpe, T., Caudron, C., et al. (2022). Spatial and temporal variability of underwater gas seepage in the Laacher See volcanic lake, Eifel Volcanic Field, Germany. 101th Journées Luxembourgeoises de Géodynamique (Luxembourg).”

As a result, in the legend of Figure 1, where

“The purple square represents the hydrophone location inside the fiber optic loop. Each red dot depicts a CO2 gas seep identified by Goepel et al.^38^. The high-resolution bathymetric map is from Goepel et al.^38^. Map is generated with Cartopy v0.22.0^40^.”

now reads,

“The purple square represents the hydrophone location inside the fiber optic loop. Each red dot depicts a CO2 gas seep identified by Albers et al.^38^. The high-resolution bathymetric map is from Albers et al. (2022)^38^. The map is generated with Cartopy v0.22.0^40^.”

The original Article has been corrected.

