# Supplementary material for: Monitoring underwater volcano degassing using fiber-optic sensing
Source: Sci Rep. 2024 Feb 7;14:3128. doi: 10.1038/s41598-024-53444-y (PMC10850492; doi:10.1038/s41598-024-53444-y)
Supplement: Supplementary file 1 — Supplementary Information. [file 41598_2024_53444_MOESM1_ESM.pdf]

Supplementary Materials for

Monitoring Underwater Volcano Degassing Using Fiber-Optic Sensing

Corentin Caudron *et al.*

*Corresponding author: Zack Spica, zspica@umich.edu

This PDF file includes:

Figs. S1 to S9

Other Supplementary Materials for this manuscript include the following:

Movies S1

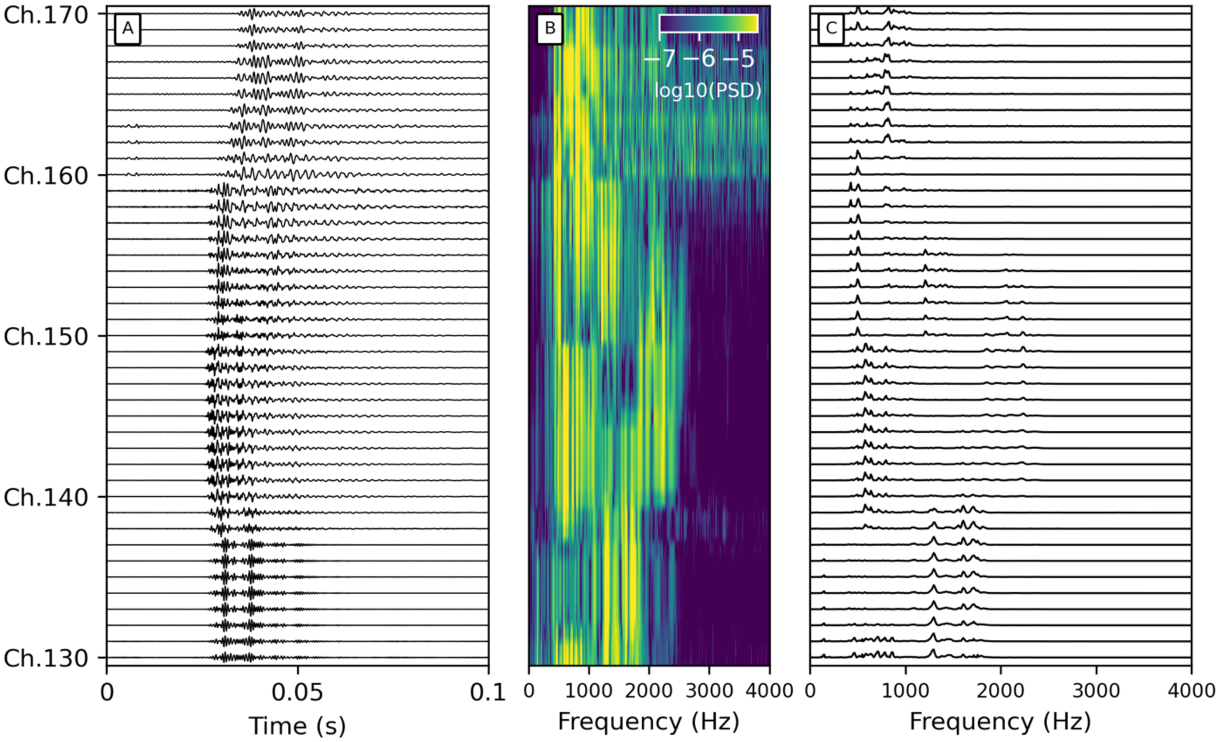

Fig. S1. Evolution of a bubble waveform along a section of the array. A) Bubble waveforms between ch.130-170 (i.e., over 40 m). B) Normalized Power Spectral Density (PSD) functions of the same bubble shown in A). Brighter and darker colors correspond to higher and lower energy, respectively (in log scale). C) Spectra of the waveforms shown in A).

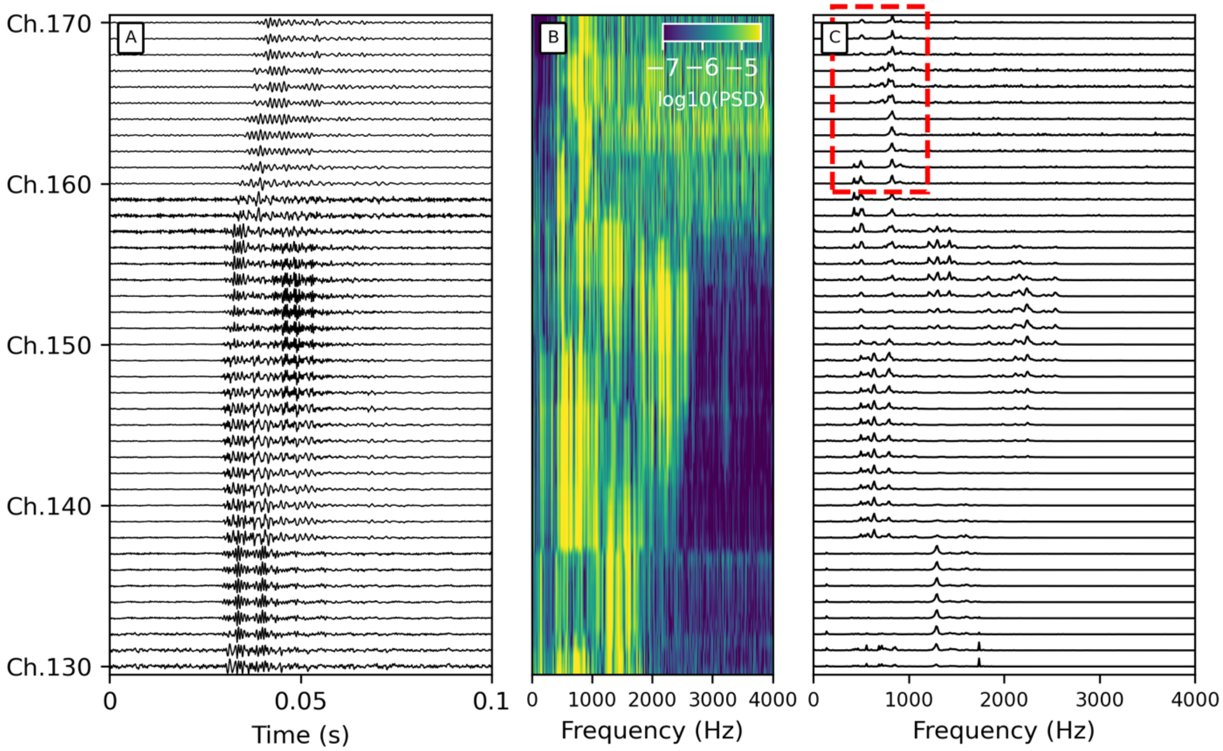

Fig. S2. Evolution of a bubble waveform along a section of the array. A) Bubble waveforms between ch.130-170 (i.e., over 40 m). Low SNR waveforms are bandpass filtered for visualization purposes. B) Normalized Power Spectral Density (PSD) functions of the same bubble shown in A). Brighter and darker colors correspond to higher and lower energy, respectively (in log scale). C) Spectra of the waveforms shown in A). The red rectangle represents the frequency band of the filter applied to selected channels in A).

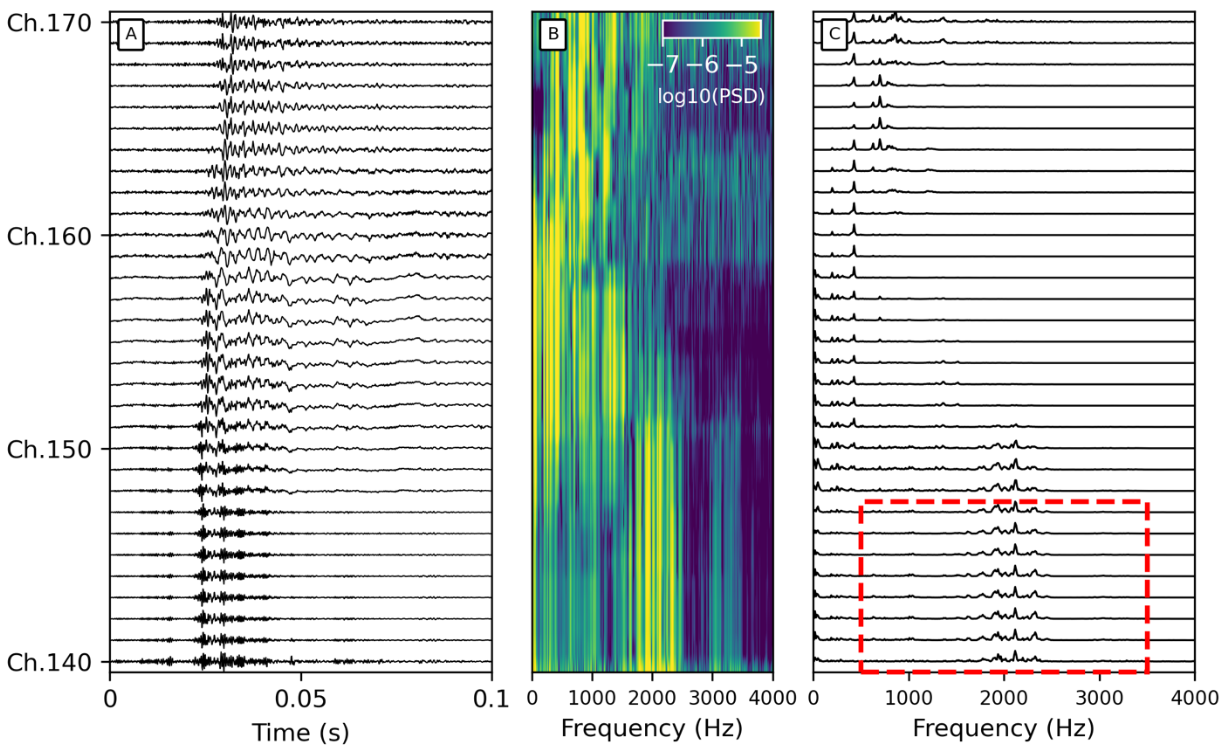

Fig. S3. Evolution of a bubble waveform along a section of the array. A) Bubble waveforms between ch.140-170 (i.e., over 30 m). Low SNR waveforms are bandpass filtered for visualization purposes. B) Normalized Power Spectral Density (PSD) functions of the same bubble shown in A). Brighter and darker colors correspond to higher and lower energy, respectively (in log scale). C) Spectra of the waveforms shown in A). The red rectangle represents the frequency band of the filter applied to selected channels in A).

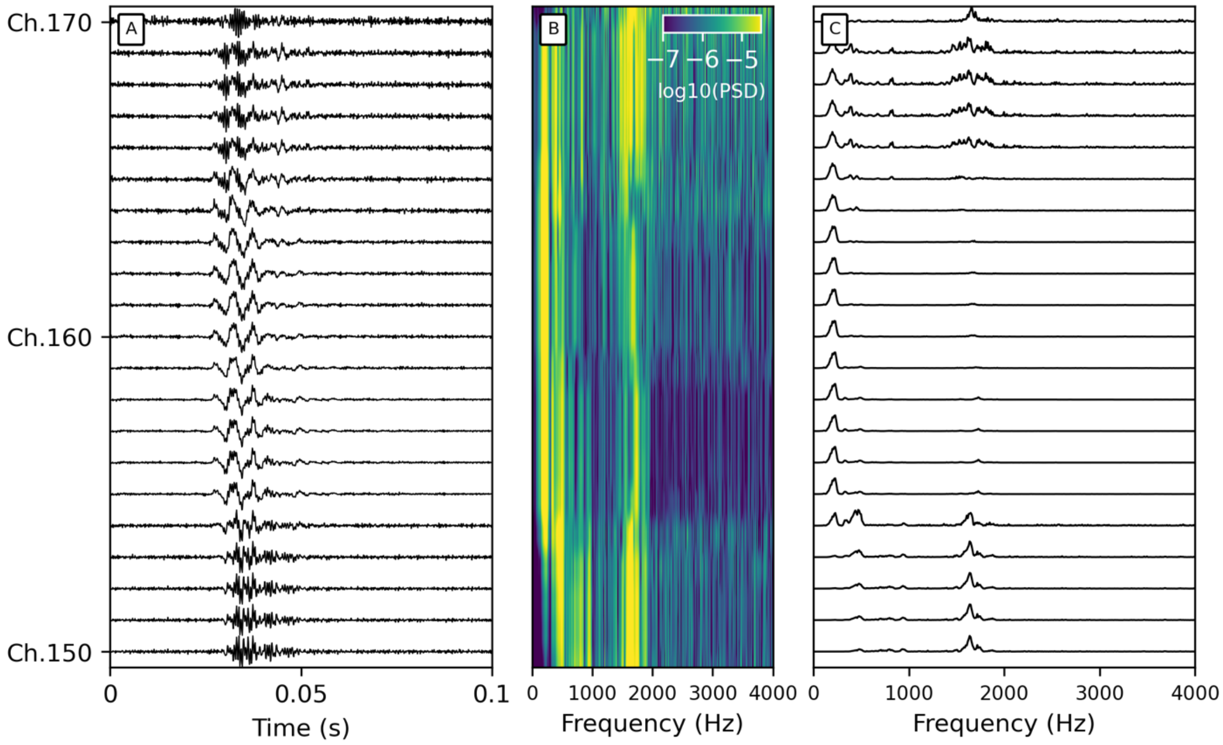

Fig. S4. Evolution of a bubble waveform along a section of the array. A) Bubble waveforms between ch.150-170 (i.e., over 20 m). B) Normalized Power Spectral Density (PSD) functions of the same bubble shown in A). Brighter and darker colors correspond to higher and lower energy, respectively (in log scale). C) Spectra of the waveforms shown in A).

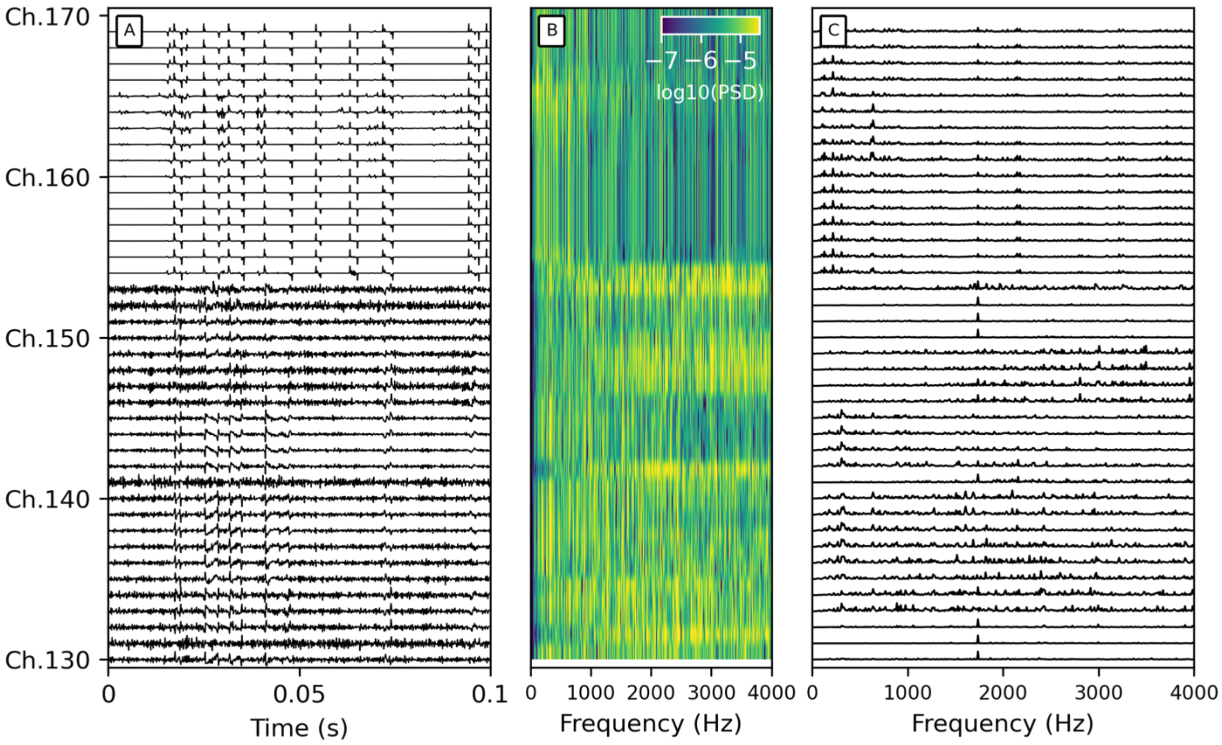

Fig. S5. An example of suspicious signal and its evolution along the cable end. A) Signal waveforms between ch.590-630 (i.e., over 40 m). B) Normalized Power Spectral Density (PSD) functions of the time-series shown in A). Brighter and darker colors correspond to higher and lower energy, respectively (in log scale).

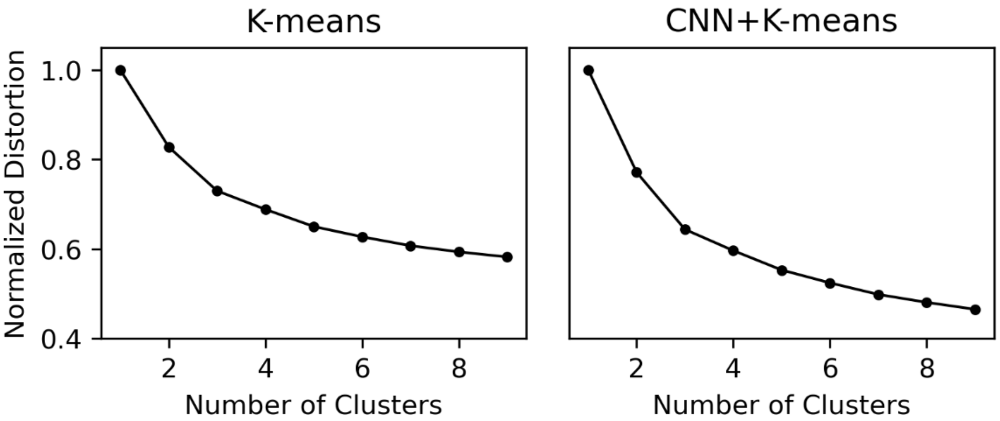

Fig. S6. Elbow test results. Left panel shows the elbow test of the K-means algorithm with original large dimension spectrograms as the input. Right panel shows the elbow test of the K-means algorithm with reduced dimensionality matrices by CNN as the input. A relevant elbow point corresponds to three or four clusters.

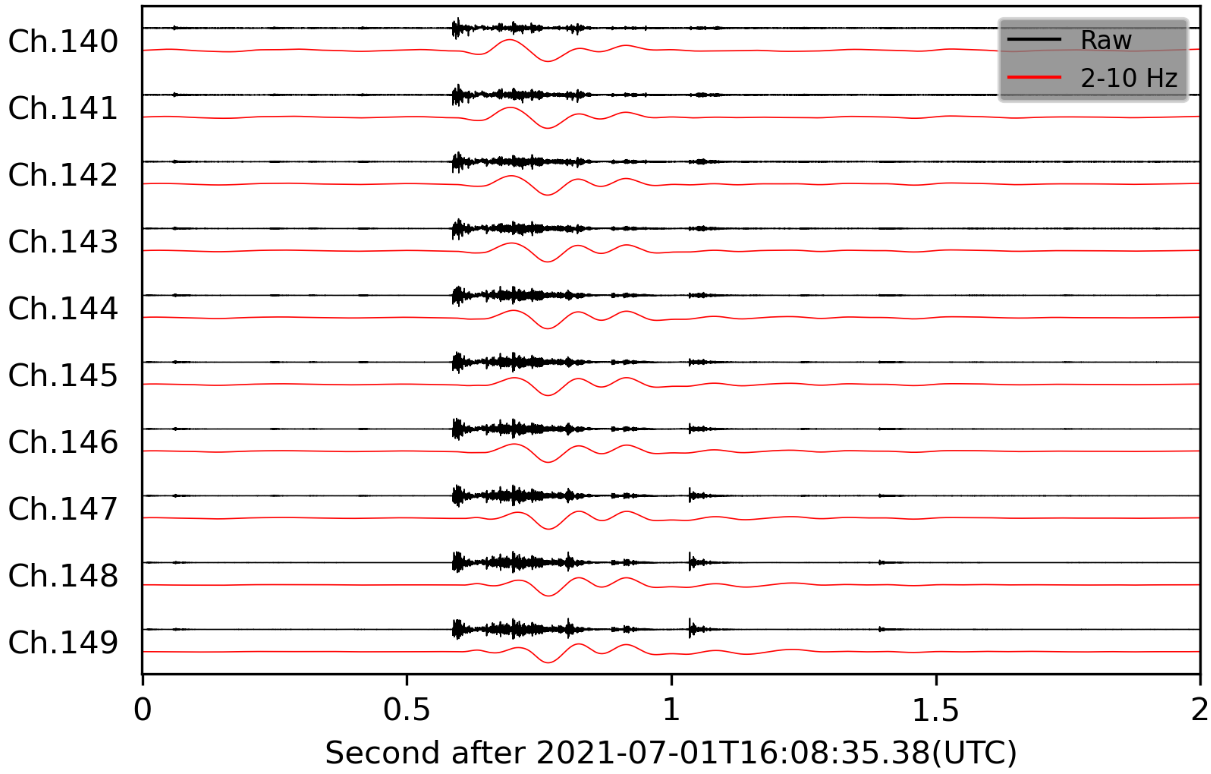

Fig. S7. Waveforms of a large bubble along ch.140-149. Black traces are unfiltered waveforms and red traces are waveforms bandpass filtered between 2-10 Hz for each black waveform above them, respectively.

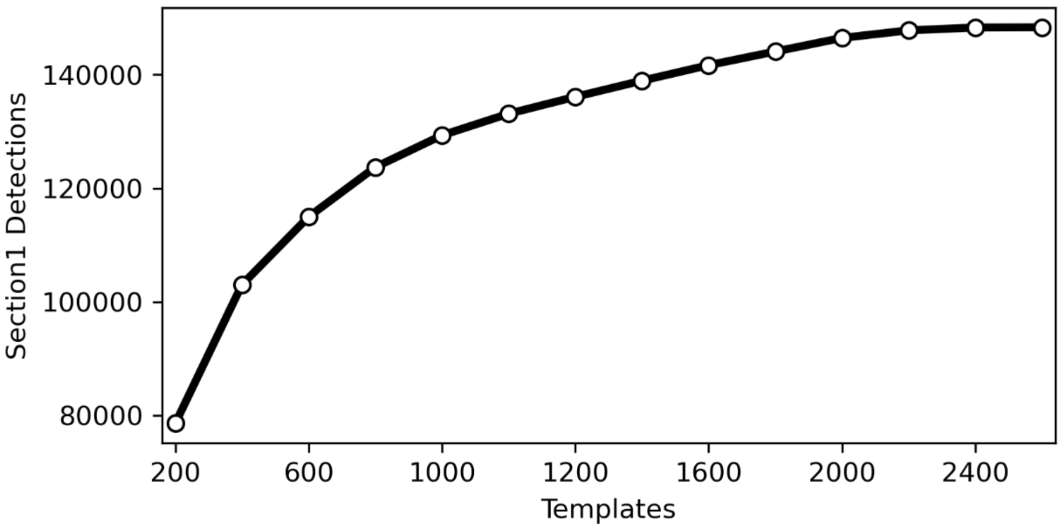

Fig. S8. Template matching results of Zone 1 by small batches of templates. Detection numbers are represented as a function of the number of templates used. We observe a general trend of diminishingly increasing and a tendency of convergency.

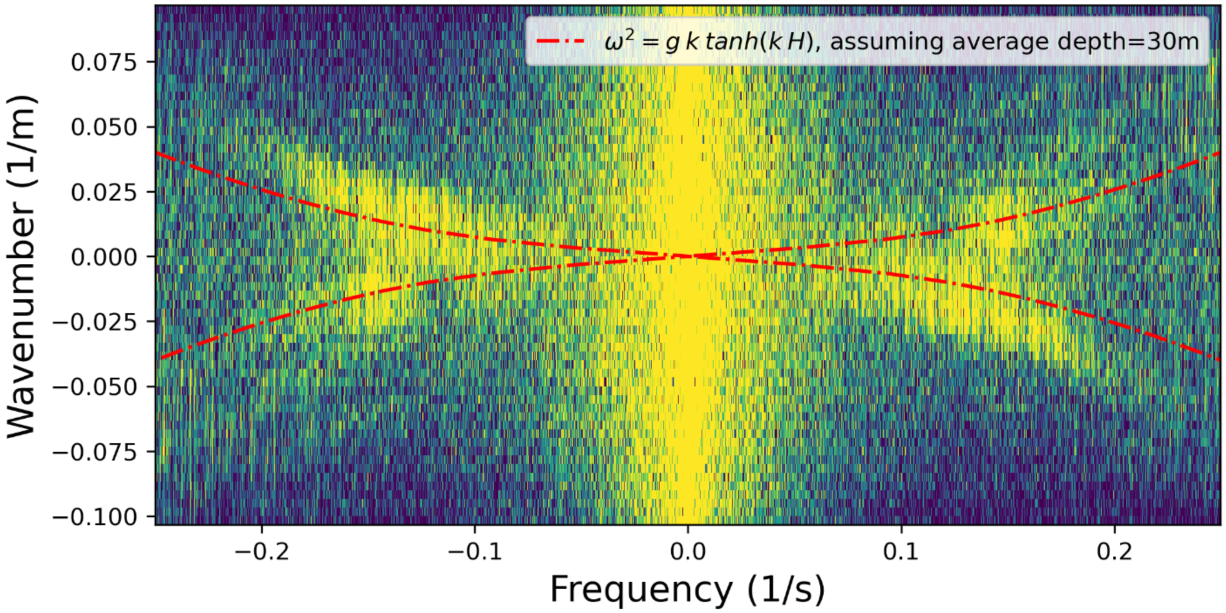

Fig. S9. Frequency-wavenumber spectrogram from channel 240-300 showing wave propagation in low frequency domain (< 0.25 Hz). Spectrogram is stacked with ~18 hours of data. The red dotted curve shows the theoretical dispersion relation of surface gravity waves, assuming a 30m average depth of the subsection.

Movie S1. (Separate file)
A video acquired with a smartphone close to the interrogator. The section of the cable underwater corresponds to channels 140 to ~170.
